# Factors Affecting Vaccine Attitudes Influenced by the COVID-19 Pandemic

**DOI:** 10.3390/vaccines11030516

**Published:** 2023-02-23

**Authors:** Jessica D. Altman, Dashiell S. Miner, Abigail A. Lee, Aaron E. Asay, Bryce U. Nielson, Agnes M. Rose, Kaitlyn Hinton, Brian D. Poole

**Affiliations:** Department of Microbiology and Molecular Biology, Brigham Young University, Provo, UT 84602, USA

**Keywords:** vaccine hesitancy, childhood vaccines, COVID-19

## Abstract

The development of vaccines has significantly contributed to the success of disease prevention. However, there has been a sharp decline in immunization rates since COVID-19 spread globally. Seemingly overnight, the world shut down and most non-essential medical procedures were postponed. Since the COVID-19 vaccine became available, and the world started going back to normal these vaccine rates have not recovered. In this paper, we review the published literature to explore how convenience factors, perceived risk of vaccination, media or anti-vaccination ideals/movements, and healthcare professionals affect an individual’s compliance to be vaccinated to better understand the factors that contribute to the change in overall vaccination rates.

## 1. Introduction

Prior to the widespread use of vaccines there was a high rate of death and illness due to common viruses such as measles, flu, and smallpox. It is estimated that before 1963 there were six thousand individuals killed yearly by measles in the United States. Similarly, a rubella epidemic between 1964 and 1965 caused eleven thousand miscarriages in women and two thousand neonatal deaths. However, the common use of vaccines is estimated to have prevented 100 million cases of infection [1,2]. Vaccines have become one of the most effective and cost-efficient ways to prevent millions of deaths by inducing immunity against certain diseases such as tetanus, pertussis, rubella, measles, mumps, and diphtheria [1,2,3]. Mandatory vaccinations in schools led to improvements in herd protection and more healthy children [4,5]. Despite efforts prior to the COVID-19 pandemic, vaccination promotion endeavors failed to prevent an estimated 1.5 million deaths annually from vaccine-preventable diseases [3].

The spread of COVID-19 began in Wuhan, China in December 2019 [1]. This new virus was named severe acute respiratory syndrome coronavirus (SARS-CoV-2) with the accompanying disease named COVID-19. COVID-19 spread across the world as it infected individuals through person-to-person contact [6]. Symptoms varied greatly depending on many demographic factors, but overall, children were found to have milder cases of COVID-19 [1,7]. With the high rates of infection and death throughout the world many business and government closures began to take place [8]. 

Social distancing and stay-at-home orders were placed into effect to help prevent the spread of COVID-19 [9]. During this time, schools began closing and most non-life-threatening medical visits were canceled. These closures had a significant effect on the administration of childhood vaccinations [1,10]. The Center for Disease Control (CDC) reported that within one week of the United States declaring a state of emergency, there was a significant decline in childhood vaccination rates [9]. Similarly, the World Health Organization (WHO) reported a 70% reduction rate of routine immunizations during the beginning stages of COVID-19 [11]. Twenty-one countries across the world even showed a reduction of 90% in their vaccination rates [12]. A survey completed by WHO and the United Nations Children’s Fund (UNICEF) found that more than half of the 129 countries analyzed had moderate-to-severe declines in their vaccination rates. Reductions of this magnitude are expected to make 80 million children below the age of 1 susceptible to diseases that could be prevented by vaccines [10]. Since the pandemic there has been an increase in parental vaccine hesitancy, which is contributing to the low vaccination rates. The World Health Organization (WHO) has even gone as far as to say that vaccine hesitancy is one of the top ten leading threats to global health [8,13,14]. 

Our earlier work indicated that exposure to vaccine-preventable diseases or education about the organisms responsible for diseases can improve vaccine attitudes [15]. We hypothesized that exposure to the COVID-19 pandemic may improve vaccine attitudes, but that other factors may increase vaccine hesitancy. The purpose of this paper is to explore factors that contribute to vaccination during the age of COVID-19 and to analyze ways that those factors either improve or impair vaccination hesitancy or rates. The paper will address vaccine hesitancy as a whole for the entire population, both children and adults, and consider mainly routine (non-COVID-19) vaccines. The areas that will be focused on within this paper are convenience factors, the perceived risk of vaccination, the media and anti-vaccination ideals or movements, and the role of healthcare professionals in vaccine promotion or hesitancy. 

## 2. Convenience

Prior to the pandemic, vaccinations were seen to be at an all-time high in terms of raw numbers. The WHO Immunization Agenda 2030 detailed vaccination success until 2020. The number of infants vaccinated annually was up to 116 million, capturing 86% of all infants in the world. In the years between 2010 and 2018, 23 million deaths were avoided by providing people with measles vaccines. The World Health Organization started to make goals to reduce yellow fever outbreaks to 0 by 2026, eliminate meningitis epidemics by 2030, and even certify poliovirus eradication by 2023 [16]. However, many of these hopes would be dashed by the advent of COVID-19. 

In 2011, The WHO EURO Vaccine Communications Working Group established the criteria for vaccine hesitancy as being one of the three C’s, confidence, complacency, and convenience [17]. The first C is confidence, which is a belief that vaccination is effective, will not cause harm, and will overall benefit the recipient in preventing disease. During the COVID-19 pandemic, confidence in the vaccine was continually changing. The vaccine was a new type, was relatively untested compared to other vaccines on the market, and was subject to near-constant policy changes. With high confidence comes higher vaccination rates [18]. The second C, complacency, can be found in populations where individuals think vaccination is not needed or the disease is not severe enough to warrant getting vaccinated. Higher vaccination rates are present in areas where individuals have less complacency and more urgency to be vaccinated [17]. The third C, convenience, can encapsulate many different aspects of hesitancy. It can refer to the direct, indirect, and opportunity cost that people must consider before being vaccinated. Convenience due to the lack of access that people in lower- and middle-income countries (LMICs) have contributes to lower vaccination rates [1]. Convenience could refer to clinics and their ability to provide vaccinations in a time of limited resources. Additionally, convenience, in combination with confidence and complacency, can be affected by the ease of accessibility of information that people need in order to reach the decision to be vaccinated. All of these different aspects were affected in some way during the COVID pandemic. 

### 2.1. Direct and Indirect Costs of Vaccines

Convenience can be best created when direct costs and indirect costs are minimized. Direct costs are seen as the direct monetary value that one pays for a vaccine. Indirect costs are any external costs that are necessary to be vaccinated (i.e., travel costs, money lost from not working, and competing priorities). To successfully make vaccination options convenient there needs to be both an option for minimal direct and indirect costs [18]. 

Direct costs must be considered in order to balance a high demand while still maintaining an economically feasible model. A review of several pandemics in the past produced data that reflected the importance of the price of a vaccine. A study performed in Poland, Sweden, and the Netherlands showed no change in intended vaccination uptake in response to an increase in vaccine price. This may be explained by the belief that the benefits of disease prevention outweigh all costs of getting vaccinated [19]. Another study suggested that making vaccines free will best help prevent the spread of disease amongst vulnerable populations [19]. Another study suggested that making vaccines free was not economically feasible, and charging some fee was necessary to continue vaccine programs [19]. It should also be noted that a cheap vaccine may raise questions about the safety and efficacy of a vaccine [19]. Thus, how to best address direct costs for vaccines is a necessary consideration for individual countries as they consider their plan to vaccinate others. 

In addition to direct costs, patients often evaluate the indirect costs necessary to receive a vaccine. Studies have shown that conveniently located vaccine clinics or short wait times had an impact on vaccine uptake during the COVID pandemic [19]. Another study showed that physicians in urban areas were much more affected than physicians in suburban areas in regard to giving routine vaccinations during the COVID pandemic [20]. Thus, it is possible that vaccine uptake in suburban areas is higher, to reflect the convenience of having to wait less to receive a vaccine. 

Vaccine clinics could also encourage vaccination by coupling COVID vaccinations with routine vaccinations. A recent study of teenagers and their parents during the pandemic suggested that of all the teenagers that received the COVID-19 vaccination, 70% of the parents would allow their teenager to receive another routine vaccination at the same time. For the parents that would not allow their teenagers receive routine vaccinations, the most common reason was that they had already received their routine vaccinations [21]. It is therefore possible to increase routine vaccine uptake by offering multiple vaccines at once. 

### 2.2. Vaccination Campaigns in Lower- and Middle-Income Countries (LMICs)

Toward the beginning of the COVID-19 pandemic, WHO began to advise the postponement of vaccination campaigns for other diseases because of social distancing [1]. The Global Polio Eradication initiative also advised the suspension of polio vaccination programs until the second half of 2020. As a result, vaccination programs in 68 countries were disrupted, affecting around 80 million children. A total of 46 poliovirus campaigns in 38 countries were suspended and 90 vaccination programs overall were postponed [1]. Vaccination campaigns ceased for measles, polio, diphtheria, pertussis, tetanus, and meningitis. Despite these orders, WHO continued to encourage routine vaccination as best as possible [1].

In the following months, several outbreaks and reappearances of diseases emerged. Measles outbreaks were reported in 18 countries, a polio outbreak in Niger, and polio cases were reported in Chad, Afghanistan, Pakistan, Ethiopia, and Ghana. The emergence of certain diseases can illuminate the lack of generalized vaccination in certain areas, like a canary in a mine. Illnesses such as measles, mumps, and whooping cough can act as alarms for lower vaccination rates since the extremely infectious disease necessitates a high herd immunity, around 90–95% [1]. One study showed that for every death that occurred because of COVID infection during a routine vaccine clinic visit, 84 deaths in children could be prevented by maintaining childhood vaccination in Africa [22].

When advising international vaccine operations, organizations such as WHO and GAVI face significant barriers to program implementation. It is very difficult to integrate vaccination campaigns in different governments of different healthcare systems [18]. For example, LMIC governments have less political and economic power to mobilize broad vaccination campaigns [1]. Additionally, COVID caused a worldwide economic recession, compromising funding from LMICs and other programs that provide vaccination for those countries [23]. Thus, an effective program will need to be specialized for each country or region that it is used in.

### 2.3. Misinformation or Lack of Information 

Misinformation and lack of information have played a substantial role in affecting attitudes about vaccination during the COVID pandemic [24]. According to one source, the speed of transmission of false information about COVID-19 proved to be more dangerous than the speed of the spread of the virus [1]. Another study demonstrated that of the three C’s of vaccine hesitancy, confidence and convenience were positively correlated [25]. Decreasing vaccine misinformation will positively benefit confidence, convenience, and complacency toward vaccination. 

Lack of information has also proven problematic in less nefarious ways. For example, as the restrictions of the quarantine were lifted, some people were unaware that clinics were open and did not return to receive routine vaccinations that were missed during that time [26]. In a study of migrants in Europe during COVID, low literacy, language barriers, lack of vaccine information, and lack of interpreters were all barriers to routine vaccination uptake [27]. With little information about routine vaccinations and improper translating resources, people who migrate or immigrate may have less information to make a decision about vaccination. Facilitators of routine vaccine uptake in these situations would include increased cultural competence, integration, engagement, and increased access points [27]. 

### 2.4. Other Barriers to Vaccination

As international quarantines began to take effect in March 2020, many clinics and vaccine resources shut down. A study in Canada found that during the first wave 4.6% of clinics were temporarily closed, 26.3% had vaccine services postponed, and the in-person visits dropped from 99% to 18% [20]. In addition to restricted access to clinics, some people may not have been able to attend their routine vaccination clinic due to having COVID-19 [1]. For others, it is possible that the message to stay home overwhelmed the message that immunizations were supposed to continue as normal [28]. 

With the disruption of the quarantine and a large burden on the clinics that remained open, clinics began to prioritize routine vaccinations for children under 24 months old [29]. Alternatively, others suggest vaccinating adults and children where they are, such as schools, work, home, and prison [26]. However, this could be potentially problematic, especially where school and work were moved to remote access [20]. In fact, when asked about the top barriers to routine vaccination in Canada, the highest barrier was attributed to school closures and the increased difficulty to reach children at home [20]. 

## 3. Perception of Risk

Risk perception is defined as a person’s ability to process information, react, and make decisions during dangerous or risky events [30]. Perception of risk affects both confidence and complacency. The release of SARS-CoV-19 vaccines has caused people across the globe to reconsider and alter their perception as it pertains to receiving vaccinations. Different studies have shown that for some, the pandemic led to increased intent to vaccinate, while for others, vaccine hesitancy had increased. This is an area that needs further investigation to understand the differences between these reactions and how best to encourage vaccine acceptance. 

For example, from January 2020 to September 2020 COVID-19 vaccine acceptance dropped from 70% to less than 50% in Europe [31]. There are two main groups that presented in 2020 regarding those that were opposed to getting the new vaccine: those that distrusted the government and those that saw the expedited vaccine development as unsafe and unpredictable [31,32]. However, both groups of people decreased in their acceptance of the vaccine due to their concerns about vaccine safety and effectiveness of the vaccine along with general perceived risks [31]. The question that now needs to be answered is if this perceived risk of the SARS-CoV-19 vaccine has influenced the risk people now associate with other, routine vaccinations.

General vaccine hesitancy and risk perception have largely been monitored through surveys asking the intentions of guardians who are responsible for deciding if their children will be vaccinated. The number of vaccines given does not serve as a reliable indicator due to the varied effects that the pandemic has caused: economic issues, stay-home mandates, and social distancing have all contributed to a drop in vaccines administered [33]. Goldman et al. looked at how likely caregivers were to vaccinate their children against the flu following the SARS-CoV-19 pandemic. The results showed that of the 2422 participants, 54.2% planned on vaccinating their child the following year. This was an increase of 15.8% compared to the previous year. In addition, of the 1459 caregivers that opted to not vaccinate their children against the flu the previous year, 28.6% plan on vaccinating their children the next year. Additionally, only 38 of the 2422 participants (1.6%) vaccinated their child the previous year but opt not to do so the following. Two of the three largest indicators of predicting whether a parent would make the change to vaccinate their child included both the caregivers’ vaccine history and the caregivers’ concern that the child had contracted COVID-19 [34].

Another study completed in Saudi Arabia compared caregiver’s hesitancy about SARS-CoV-19 vaccines to hesitancy about routine vaccinations given mostly to children. Of note, there seems to be a large increase in the hesitancy rate of routine childhood vaccines compared to a previous, similar study completed in 2019. The 2019 study identified 20% of the population as vaccine hesitant while 45.3% of the population was identified as vaccine hesitant after the pandemic [8,35]. The 2019 study cited risks associated with the vaccine as the most common reason for vaccine hesitancy [35]. Comparatively, Temsah et al. identified that following the pandemic, 45.1% of the total population were anxious about serious adverse effects of routine vaccines for children while 70.2% percent of all participants were anxious about the adverse effects of the COVID-19 vaccine [8]. This shows a dramatic increase in overall general vaccine risk perception among caregivers in Saudi Arabia. In addition, it was seen that the population in Saudi Arabia has become more skeptical of newer vaccines and that 53.5% of the population believes newer vaccines carry more risk than older vaccines [8]. This could pose issues of acceptance as new vaccines are developed and administered.

A survey based at Children’s Hospital Los Angeles explored how guardians’ perspectives on routine vaccinations have changed because of the pandemic. Results show that guardians’ intentions to vaccinate their children have remained constant, but vaccine hesitancy has increased. These hesitations are mostly perpetuated by a guardian’s “risk perspective” or the risks that they associate with vaccination. Specifically, parents are most concerned with vaccine safety profiles [36].

One consequence of a pandemic is the effect it has on mental health. Lockdowns, social distancing, and the fear associated with contracting a potentially life-threatening disease can lead to many issues such as stress, anxiety, and depression [37]. These mental health challenges have the potential to influence a person’s risk perception in the long term. For example, it was discovered that the SARS-CoV-19 pandemic has caused many people to develop generalized anxiety disorder [38]. A generalized anxiety disorder (GAD) is defined as an “excessive, persistent, and unrealistic worry about everyday things” [39]. It is important to note that GAD is a condition that affects the entirety of a person and not just their perception of one issue or topic; in this case, while the pandemic may be a trigger for GAD, the effects of the GAD spread far beyond issues isolated to the pandemic. An increase in anxiety has been shown to affect decision-making, cause people to question the correctness of past and future decisions, and alter the ability to interpret risk [39]. The increase in GAD, or even anxiety in general, associated with the pandemic could contribute to altered risk perception towards all vaccines and lead to greater skepticism. The SARS-CoV-19 pandemic has affected the way people see and consider the risks of vaccines in general. There has been an increase in Google searches, and media coverage pertaining to vaccination and the risks involved with being vaccinated [40]. Whether or not this increased perception of risk associated with general vaccination will deter a larger population from receiving vaccinations has yet to be seen; however, the intentions of many guardians show the opposite and that a larger percentage of guardians are planning on vaccinating their children [34,36].

## 4. Media and Anti-Vaccination Movements

Media of all sorts has influenced public perceptions of healthcare for years, especially concerning vaccines. Given the governmental restrictions in previous years as well as a push for vaccination, healthcare is a hot topic in the media. News media has often been viewed as a reliable source of information. However, the public perception of media has been consistently declining over the past several decades. A Gallup poll reports that about 38% of individuals surveyed in 2022 have no trust in news media, a record high [41]. At the same time, an increasing number of individuals are receiving their political as well as healthcare information from social media. Social media platforms are much less regulated than news media and give a platform to a wide variety of people where both information and misinformation can be spread easily and quickly to a large number of users [42]. 

### 4.1. Antivaccination Movements

Antivaccination movements are contributing to the growing unvaccinated population using social media as well as public demonstrations to discourage people from vaccination [43]. In fact, from 2019–2020, social media pages with antivaccine tendencies increased their following by over 7 million individuals [44]. This has likely only increased as the pandemic has progressed and antivaccination groups have become more well known. Texas, USA is one location where antivaccine groups have likely had a negative effect on vaccination. Nuzhat et al. report that a population in Texas has had a decrease in childhood vaccinations between 47 and 58 percent, but it is not clear whether these decreases should be attributed to social distancing mandates, the strong antivaccination movements in Texas, or both [45]. 

The aforementioned example of childhood vaccinations in Texas is one example of a decrease in vaccination. Other individuals, however, report an increased desire to vaccinate as well as increased plans to vaccinate [34]. A Twitter trends study found that posts containing positive attitudes towards vaccination are much more common on that platform than posts with negative attitudes. Despite an increase in the number of users who express antivaccine attitudes on Twitter, more than 80% of posts with vaccine-related hashtags show positive viewpoints about vaccines [46]. There is evidence that anti-vaccine attitudes are spreading but there is also substantial evidence that pro-vaccine attitudes are increasing as well, making it difficult to determine if anti-vaccine groups are winning over more people or if viewpoints are just becoming more polarized with an increased number of people taking a side and making their affiliation known.

### 4.2. Polarization in Media

Polarization is relevant in news media as well as social media. Trust in media is already polarized when viewed from a political standpoint. The Gallup poll mentioned previously shows this polarization with survey participants, in which those who identify as Republicans are much less likely to trust news media compared to Democrats. Their survey indicates that about 70% of Democrats surveyed have at least a fair amount of trust in news media while only 14% of surveyed Republicans have a similar level of trust [41]. This indicates that viewpoints in media are resulting in a different amount of trust from these two political groups. Similar results were found in a study specific to the relationship between COVID-19 reporting and trust in media [47].

This lack of trust in media can motivate individuals to consider using other sources to supplement their intake of information. An analysis of Google search trends about vaccines correlates peaks in searches with significant milestones during the heat of the COVID pandemic [40]. This trend indicates that there was an emotional aspect towards the desire to intake information which can open up an individual to manipulation and other forms of misinformation and dishonesty [48]. The same is true of social media. Most social media platforms are tailored to the user’s profile, meaning they will see more of the same content they reacted to, or their friends and followers reacted to. This increases polarization as “undecided” individuals can be quickly exposed to polarizing viewpoints on social media and then see more and more of those viewpoints [44,49,50]. Polarizing viewpoints are inevitable, but they have been heightened as social media has become the main source of information for many, and misinformation about vaccines has flooded platforms in the wake of COVID-19.

### 4.3. Social Media as a Source of Misinformation

Before the pandemic, social media was largely unregulated, and many people took advantage of that to spread misinformation. Many individuals have cited social media as a reason for their refusal or hesitancy to receive a COVID-19 vaccine [21]. In addition to groups spreading misinformation, some is spread by individuals. These individuals simply want to share opinions but, in many cases, these opinions are unfounded and do not have evidence to support them [51]. Social networks are attempting to remove as much false information as possible but have faced resistance. Many individuals have claimed they are being silenced and many people are frustrated by what they claim is censorship [52].

### 4.4. Strategies to Correct Misinformation and Increase Vaccination

Social media can be a useful tool for spreading correct information easily to a wider population than traditional methods. Twitter as well as Facebook and other social media sites are common platforms that are used to communicate novel scientific discoveries and share essential information with a lay audience in addition to costly and inaccessible journals and publications [53]. This allows more people to receive scientific information from the source rather than a secondary news article or an inaccurate summary. Further focusing on spreading science and scientific articles through social media will allow greater access to scientific knowledge and also challenge much of the misinformation present on those platforms [50,54]. This increased spread of scientific information would allow people to make more educated decisions and likely increase vaccination trends.

Another important way to increase vaccination would be to utilize governmental programs more effectively. For example, a survey study in Saudi Arabia showed that parents were more likely to accept vaccination when parents used the Ministry of Health as their main source of information [8]. A study in Germany showed that COVID-19 vaccination was strongly associated with trust in the government [55]. With social restrictions largely absent, more emphasis should be put on notifying the public about the need to be vaccinated for more than just COVID-19 [45]. Social media is a perfect tool for this in addition to other forms of media and publications. Social media can also be used to analyze possible side-effects of vaccination as it was used during the time of the pandemic to track and document symptoms to create a more comprehensive list of said symptoms [56].

These mechanisms of addressing misinformation must respond quickly to many topics. For example, the ability of SARS-CoV-2 to mutate quickly will likely require repeated and updated vaccinations, but that reasoning is not always clear or presented well. Likewise, information about the symptoms, both short- and long-term, of COVID-19 disease should be uniformly and accurately presented. Since this information is currently being discovered, it is imperative that reasons for changes in policy when new scientific understanding is reached are clearly articulated and disseminated to the public. 

## 5. Health Care Professionals

There is a well-established link between positive vaccine attitudes in health care providers (HCPs) and an increased rate of vaccine uptake in their patients [57,58,59]. The more overall positive vaccine attitudes that a provider has, the more the provider recommends it to patients, family, and friends [60]. Since HCPs remain the strongest influencer of vaccine decisions, both before and during the COVID-19 pandemic, it follows that their views on vaccination have significant effects on patient vaccine uptake [58,61,62,63]. In a study performed with nurses before the COVID-19 pandemic, it was demonstrated that a decrease in nurse vaccine uptake correlated with a decrease in patient vaccine uptake [64]. Several other studies found that the provider’s knowledge and experience with vaccines were linked with the increased likelihood of recommendation to patients [60,65]. Taken together, these studies further support the essential role that general vaccine belief and behavior of HCPs play in influencing vaccine uptake in their patients.

Several factors affect vaccine hesitancy among healthcare workers. Increased knowledge about the COVID-19 vaccine as the pandemic progressed was linked to increased positive vaccine attitudes among healthcare workers [63]. At the same time, this effect is mediated by education level and specialty of care [66,67]. The more education a healthcare provider has, the less likely they are to be vaccine-hesitant and the more confident they feel addressing the vaccine concerns of their patients [65,66,68]. Additionally, those who work in primary care have reduced rates of vaccine hesitancy compared to those who work in medium complexity care settings or work in administrative positions [69]. Notably, though, the promotion of vaccination by superiors significantly influences vaccine uptake of their subordinates no matter the education level of the HCP [70]. Vaccine hesitancy was additionally decreased by contact with immunocompromised patients and those with COVID-19 [64,67]. Notably, the increased contact with these patients decreased general vaccine hesitancy, not just COVID-19 vaccine hesitancy [63]. Additionally, studies note that the culture and guidelines of their institution prevent addressing vaccine hesitancy among HCPs adequately [71].

In the case of HCPs that are vaccine hesitant, many studies report that there is a fundamental difference in the way they view their role in the healthcare system [72]. They do not view themselves as a role model and maintain that their personal vaccine choice does not affect the vaccine uptake of their patients [58,73]. The data, however, tell a different story. Worldwide, HCPs with positive vaccine attitudes significantly increase the rate of vaccine uptake while HCPs with vaccine-hesitant attitudes significantly reduce the rate of vaccine uptake [60,74].

Finally, the literature agrees that systemic approaches need to be taken to both decrease HCP vaccine hesitancy and increase training that teaches how to adequately respond to their patient’s vaccine concerns [73,75]. Before the pandemic, many HCPs reported that they felt that they were stretched with time during visits, had limited resources, and had a heavy workload prohibitive to obtaining increased training regarding vaccine hesitancy [58,73]. It seems, though, that COVID-19 has brought vaccine hesitancy into the spotlight, leading to a shift in priority towards increases in training [63]. Still, more training needs to be integrated into all levels and areas of care. In hospitals especially it is not common to discuss vaccine concerns, leading to a lack of skill and knowledge on the part of the HCPs [76]. Taken together, these findings indicate that while some improvements in training have been made due to the COVID-19 pandemic, there is more work to be completed. Vaccine hesitancy training needs to become integrated into all levels of care. Luckily, higher education institutions can effectively implement additional training with their students and HCPs that can have a significant effect on general vaccine uptake [67].

## 6. Discussion 

The COVID-19 pandemic had both positive and negative effects on attitudes toward common vaccinations and taught several lessons about vaccination programs. When dealing with convenience factors, travel, expense and wait times should be minimized. Parents are more likely to vaccinate their children when routine vaccinations can be given together with their COVID-19 vaccinations. Doing this would help mitigate some of the indirect costs of receiving vaccinations. During the pandemic, many closures, such as schools and doctors’ offices, made it difficult for vaccination programs to be effective. As these closures begin opening there will need to be programs set in place to catch up on immunizations.

When analyzing risk perception, it is important to consider that anxiety may drive increased vaccine hesitancy. Given the correlations between trust an individual has in government and the vaccine’s safety, the more likely that individual is to be vaccinated if this trust can be supported and improved. Additionally, risk perception is affected by misinformation in the media and on social media. There has been a surge of anti-vaccination misinformation that has increased the risk perception for vaccinations for many people. Interestingly, however, pro-vaccine messaging has also increased substantially, suggesting that using, rather than dismissing, social media may be an effective strategy to minimize vaccine hesitancy.

Healthcare workers can have a positive or negative effect on their patient’s vaccination rates depending on how much they promote vaccinations. To increase healthcare provider attitudes toward vaccinating there needs to be an increase in training and education for healthcare workers to increase their knowledge.

A common response to all forms of vaccine hesitancy is to develop and enforce laws and mandates that compel populations to receive vaccinations. These mandates are enforced by penalties, which attempt to coerce the individual into adhering to local vaccination expectations. Penalties could include financial, educational, employment, restrictions, or loss of liberty [77]. One study showed that these restrictions on participation did not result in an increase in vaccination, only in an avoidance of these activities by those experiencing vaccine hesitancy [78]. Vaccine mandates in children are historically effective and should be continued. There is evidence that mandates for COVID-19 were effective in many settings. However, mandates also present the possibility of increased resistance, especially in those that do not trust authority already [79]. Mandates seemed to be less effective in the adult population during the COVID-19 pandemic, especially compared to vaccine access and communication about vaccine function [79]. They should continue to be included in an overall vaccination strategy but should not function as the only part of that strategy.

Multiple factors affect whether or not an individual will have routine vaccinations, and many of these have been affected by the COVID-19 pandemic. As the world gets back to a new normal, post-pandemic, there needs to be consideration taken for how childhood vaccination rates can rebound to pre-pandemic levels or higher. Some strategies include increasing convenience, effective use of social media and governmental resources to dispel misinformation, and focused training of healthcare workers to encourage vaccination.

## Data Availability

All data is included in the article.
